# FlyAtlas 2: a new version of the *Drosophila melanogaster* expression atlas with RNA-Seq, miRNA-Seq and sex-specific data

**DOI:** 10.1093/nar/gkx976

**Published:** 2017-10-24

**Authors:** David P Leader, Sue A Krause, Aniruddha Pandit, Shireen A Davies, Julian A T Dow

**Affiliations:** Institute of Molecular, Cell and Systems Biology, College of Medical, Veterinary and Life Sciences, University of Glasgow, Glasgow G12 8QQ, UK

## Abstract

FlyAtlas 2 (www.flyatlas2.org) is part successor, part complement to the FlyAtlas database and web application for studying the expression of the genes of *Drosophila melanogaster* in different tissues of adults and larvae. Although generated in the same lab with the same fly line raised on the same diet as FlyAtlas, the FlyAtlas2 resource employs a completely new set of expression data based on RNA-Seq, rather than microarray analysis, and so it allows the user to obtain information for the expression of different transcripts of a gene. Furthermore, the data for somatic tissues are now available for both male and female adult flies, allowing studies of sexual dimorphism. Gene coverage has been extended by the inclusion of microRNAs and many of the RNA genes included in Release 6 of the *Drosophila* reference genome. The web interface has been modified to accommodate the extra data, but at the same time has been adapted for viewing on small mobile devices. Users also have access to the RNA-Seq reads displayed alongside the annotated *Drosophila* genome in the (external) UCSC browser, and are able to link out to the previous FlyAtlas resource to compare the data obtained by RNA-Seq with that obtained using microarrays.

## INTRODUCTION

For almost ten years the FlyAtlas database (1) and web application (2) (hereafter referred to as ‘FlyAtlas 1’) has provided research workers with information regarding the expression of genes in the tissues of *Drosophila melanogaster*, one of the most important model eukaryotic organisms. The *Drosophila* genome contains many genes homologous to ones in *Homo sapiens*, including 75% of those known to be involved in human disease (3). The importance of FlyAtlas is indicated by the large number of literature citations it has received (∼1200 at the time of writing).

Despite the great utility of the FlyAtlas resource, the technology on which it is based—hybridization to Affymetrix microarrays (4)—has limitations: most notably that it does not differentiate between the extent of expression of different mRNA transcripts for the same gene, and that the probe sets are frozen in time, based on the original (2000) release of the *Drosophila* genome. The subsequent introduction of RNA-Seq (5) has provided a convenient means of overcoming this limitation, and we have now utilized this to update the facility to ‘FlyAtlas 2’, which is described here. In addition to providing information on gene transcripts, the RNA-Seq approach has eliminated the previous ambiguity for genes for which the microarray probe sets turned out not to be unique (2), and has produced data with the capacity to be reprocessed to accommodate future revisions of the *Drosophila* reference genome.

In undertaking this new work, we were able to address some other limitations of the original: the lack of information for *Drosophila* microRNAs (6), the absence of separate data for the somatic tissues of male and female flies, and the unsuitability of the web interface for viewing on the mobile devices that have since become ubiquitous.

## METHODS

### Biological

As previously for FlyAtlas 1, the insects used for FlyAtlas 2 were wild-type *D. melanogaster* of the Canton S strain. The adults were reared at 23°C in a 12 h:12 h light:dark regime on standard *Drosophila* diet, and sacrificed 7 days after adult emergence. The larvae were third instar feeding larvae, raised under the same conditions, and sampled before the wandering stage. The tissues were dissected as previously described (1,2). The tissues were transferred to 500 μl Qiazol (Qiagen) after dissection from the animal and stored at –80°C until enough tissue had been collected—normally sufficient to yield at least 200 ng/μl total RNA. Supplementary Table S1 shows the average number of flies dissected for different tissues to yield this amount of RNA for each of the three biological samples.

### Isolation of RNA

The tissue/Qiazol samples were thawed and vortexed for 30 s to ensure that the tissues were disrupted. Mechanical force was applied for certain tissues (e.g., carcass) because they did not completely break down in the Qiazol. The samples for individual triplicates were pooled and then dispensed into 1 ml aliquots. Chloroform (200 μl) was added to each tube, which was then vortexed again for 30 s. The aliquots were incubated at room temperature for 3 min and subjected to centrifugation at 4°C for 7 min at 12 000 × *g*. The upper phase was removed, transferred to a fresh tube, and stored on ice. The remaining Qiazol solution was extracted again with chloroform as above. The Qiazol solution was removed and samples pooled for one further chloroform extraction. The upper aqueous phase was collected and combined with the previously saved aqueous phase. Ethanol (100%—1.5 volumes) was added and the RNA purified using a Qiagen miRNeasy mini kit according to the manufacturer's instructions. The optional DNase step, the optional drying of the column, and back-elution were all included.

The concentration of RNA was determined using a ThermoFisher ND-1000 NanoDrop Spectrophotometer. The quality of the RNA was determined using an Agilent Bioanalyzer according to the manufacturer's instructions.

### RNA-Seq

Separate libraries were prepared for total RNA or microRNA sequencing by Edinburgh Genomics on an Illumina HiSeq 2500 System, using paired-end chemistry (125 nt reads) in the case of total RNA. For both the total RNA and microRNA sequencing at least 200M reads per lane were performed.

### Computational analysis

Total RNA was analysed using the Tuxedo pipeline (7) and an Ensembl version of the *Drosophila* Release 6 reference genome (8). At the time of writing this was Ensembl BDGP6, provided by Illumina and dated March 2016. It is intended to update this annually and current version details can be found in the Docs section of FlyAtlas 2, in the section ‘FlyAtlas 2 & Third-party Data…’ under ‘Version Information’. MicroRNA was analysed using CapMirSeq (9).

### Database

The processed data were in the form of FPKM (Fragments Per Kilobase of transcript per Million mapped reads), or RPM (Reads Per Million) in the case of microRNAs. They were used to populate a MySQL relational database (FlyAtlas2). The schema for this shown in Supplementary Figure S1 and the table attributes in Supplementary Table S2. The gene information that the database includes is from *Drosophila* Release 6 reference genome, as indicated above, with additional symbol and name information from FlyBase. The gene ontology information was derived from a combination of file of GO ids and name descriptors from the Gene Ontology Consortium (www.geneontology.org) and a file of GO ids corresponding to individual genes from FlyBase (flybase.org). Full details and version dates can be found in the Docs section of FlyAtlas 2, as above. It is intended to update this annually—at the time of writing the files used were from April to June 2017.

### Web application: technical

The FlyAtlas 2 web application utilizes a Java servlet to generate web pages and communicate with the relational database. Java packages from the Apache Commons Mathematics Library (http://commons.apache.org/math/) were included for statistical calculations. As previously, separate custom servlets provide the functionality for text-entry auto-suggest and for menu-population features. The application makes use of client-side pure JavaScript, including an external script by A. Tipson (https://gist.github.com/dtipson/7401026) that enables the ‘Request Desktop Site’ option on the mobile Safari web browser.

## VALIDATION OF DATA

The original data obtained from microarray hybridization experiments for the expression of genes in different tissues were validated by reference to certain previously studied genes with extreme specificity of expression (Table 1 of (1)). A similar analysis performed on the present data obtained by RNA-Seq provides similar validation (Supplementary Table S3).

The database for FlyAtlas 2 contains data not present in that for FlyAtlas 1 (flyatlas.gla.ac.uk/index.html): separate data for gene expression in male and female flies, and information on individual RNA transcripts. An indication of the validity of these data is given in Supplementary Figure S2, which presents results for the well-studied genes, *tra, dsx* and *fru*, involved in the sexual differentiation of *Drosophila*, and known to exhibit sexual dimorphism for different transcripts in somatic tissues (10). The results show differences between males and females that are consistent with the established sex-specific splice sites.

To obtain an indication of the validity of our data on the expression of *Drosophila* microRNAs we focussed on some of the published data that appeared most comparable to our own. This was the work of Fagegaltier *et al.* (11) on expression in the ovaries and testes of adult *Drosophila*, albeit 2–4 days old, rather than 7 days old as in our work. Supplementary Table S4 compares microRNAs that are highly enriched in these tissues, and shows considerable similarity between the two studies, despite some marked differences. These similarities (and also others not shown for tissues such as brain) gives us confidence in the validity of our data and suggest that the differences are likely to have a biological cause (age, mating status, diet etc.). Users of FlyAtlas 2 are advised, however, that such qualitative and quantitative differences exist.

Finally in relation to the general validity of our data we would mention two other points. First, for many genes that do not exhibit sexual dimorphism there is good consistency between the triplicate results for male and female flies. Second, in many genes that are not expressed in a stage-specific manner tissue-specific expression is observed in both adults and larvae. This adds to our confidence in the data.

RNA-Seq analysis of a range of *Drosophila* tissues was also performed in the modENCODE project (www.modencode.org) but the generally different tissues and insect stages used there mean that the two studies generally complement, rather than duplicate, one another, and comparison was less useful for validation purposes.

We were interested in how well the RNA-Seq data from FlyAtlas 2 correlated with the microarray data of FlyAtlas 1. In brief, there is generally good correlation for genes that are highly expressed or enriched in a particular tissue—albeit not perfect. The correlation is poorer for weakly expressed genes. To illustrate this point, we considered Malpighian tubules (cf. Table 2 of (1)), and compared the 60 genes that showed the greatest enrichment in the microarray and the RNA-Seq data (Supplementary Table S5). Thirty-nine of these genes were found in both sets. In most of the other cases the genes showing a discrepancy were either highly expressed in tubule but had a higher ‘whole fly’ baseline in one set, or their expression was sufficiently low in both cases to place low reliance on the value of enrichment.

In general discrepancies between FlyAtlas 1 and FlyAtlas 2 in weakly expressed genes is hardly surprising. In RNA-Seq one can examine the sequence reads in the context of the genome map to eliminate false positives, although false negatives cannot be excluded in this way.

## SUMMARY OF DATA & IMPROVEMENTS FROM FLYATLAS 1

As already mentioned, the most important changes in the FlyAtlas 2 database are the inclusion of data for the expression of individual transcripts, for the expression of microRNAs, and of separate data for male and female flies in adult somatic tissues. More recent versions of external databases have been employed and the genes for which data is ambiguous have been markedly decreased in number.

The genes and transcripts present in the FlyAtlas 2 database are summarized in Table 1. It can be seen that, in addition to the increased number of genes from FlyBase that only encode RNAs (very few in FlyAtlas 1) there are 763 more protein-coding genes—the result of employing the extensively revised *Drosophila* reference genome, Release 6.

**Table 1.** Genes and Transcripts represented in the FlyAtlas 2 database

	FlyAtlas 2			FlyAtlas 1		
	FlyBase^a^	FlyAtlas^b^	Signal^c^	FlyBase^d^	FlyAtlas^e^	Signal
Total Genes	17 556	17 485	14 205	13 251	12 534	12 303
Protein-coding	13 970	13 899	12 506	13 207	12 490	12 276
RNA-coding	3 586	3 586	1 828	44	44	27
Total Transcripts	34 715	34 142	24 340	—	—	—

^a^ *Drosophila melanogaster* Release 6, reference genome. All these genes were deposited in the Gene table (Supplementary Figure S1) of the FlyAtlas 2 database.

^b^Genes or transcripts in the FPKM or RPM output of the computational analysis of sequence reads.

^c^A signal was regarded as an FPKM greater than 2 or an RPM (microRNAs) greater than 100 in at least one tissue.

^d^ *Drosophila melanogaster* Release 3, reference genome

^e^Genes for which there is at least one unique microarray probe-set.

It has previously been explained that values for the expression of 717 of the genes in FlyAtlas were ambiguous because certain microarray probe sets hybridized to transcripts other than those for which they had been designed (2). Most of these ambiguities have been resolved in FlyAtlas 2. However, 71 genes in *Drosophila* reference genome, Release 6, were absent from the FPKM or RPM output of the computational analysis of the sequence reads. The reason for this is that analysis of the expression of genes by RNA-Seq measures expression of RNA transcripts—not their protein products—and in these cases the transcripts for the ‘missing’ genes are identical to those for an overlapping gene. Such an example of a pair of genes with an identical transcript is DNApol-γ35 and GatC. This mRNA transcript is dicistronic, and two different translational start sites are employed to produce completely distinct proteins. Users searching for any of the 71 ‘absent’ genes (listed in Supplementary Table S6) are informed of the situation in the screen output, as are users who search for any of the 68 genes present in the database that have identical transcripts.

It can be seen from Table 1 that a related situation occurs with transcripts, 573 being absent from the output (listed in Supplementary Table S7). There must clearly be additional reasons for this besides mRNAs with multiple translational start sites, and it turns out that many individual RNA transcripts have been given two or more FBtr identifiers to reflect multiple protein products caused by read-through of a termination codon. An example is discussed below in relation to the transcripts of the gene used to illustrate the search facility of the web application.

Of the 17556 genes from FlyBase in the FlyAtlas 2 database, signals for approx. 81% were detected in at least one tissue. This compares with signals for ∼93% of genes detected in FlyAtlas 1, although for protein-coding genes the difference is much smaller: approx. 90% v. 93%. This suggests a lower expression of the transcripts of RNA genes than protein genes, but to some extent this reflects the fact that tRNAs and one class of snoRNA are too small to be detected by the total RNA sequencing methodology. In addition, FlyBase classes the precursors of the microRNAs as transcripts, although only the mature 3′ or 5′ portions of the processed precursor stem-loop are detected in our work. Ribosomal RNAs are also excluded. Explanations why some genes are not detected in FlyAtlas 2 include expression only in tissues not yet examined or in embryonic and pupal stages. The numbers of each class of RNA detected are listed in Supplementary Table S8.

Data for the expression of individual transcripts were not available for FlyAtlas 1. For FlyAtlas 2, ∼70% of the 34 715 transcripts were detected in at least one tissue.

One other aspect of the database has been improved in FlyAtlas 2—the gene ontology data from www.geneontology.org and FlyBase have been updated, enhancing the ‘Category’ searches which employ them. It is intended to update these data at least annually.

## DESCRIPTION OF THE WEB APPLICATION & IMPROVEMENTS FROM FLYATLAS 1

The user interface to the web application is based on the later version of FlyAtlas 1 (2), aiming to continue the emphasis on simplicity while presenting the new data on male and female flies and individual transcripts. There are two other main new features—a download facility and the option to view reads in the external UCSC browser. These are implemented in a manner that does not complicate the interface and, in fact, the responsive nature of the HTML and CSS coding provides a simpler view on mobile devices.

The three main ways of searching are by ‘Gene’ identifier, by ‘Tissue’, and by functional ‘Category’. The tabular output from these three search modes is similar in format, so we shall focus on the description of ‘Gene’ searches, which constitute almost half the usage of FlyAtlas 1.

**Figure 1.**
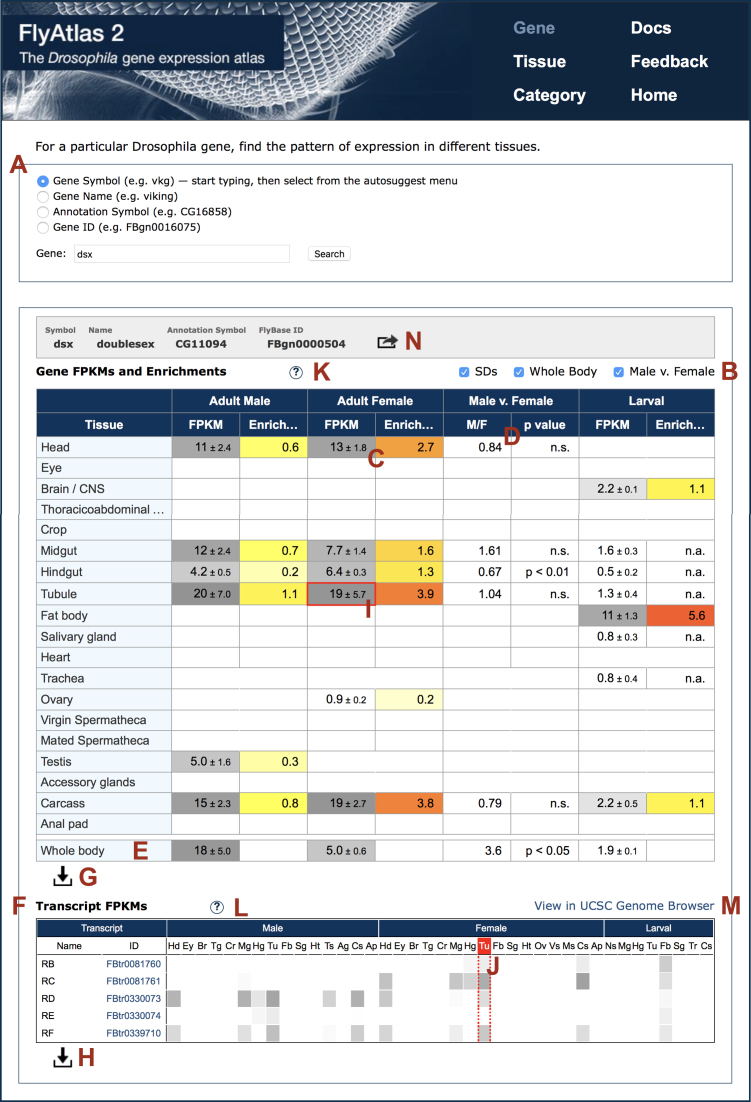
Results page returned from a ‘Gene’ search in the FlyAtlas 2 web application. The items indicated alphabetically are (**A**) entry form, (**B**) ‘tick’ (‘check’) boxes for optional display features, (**C**) standard deviations, (**D**) male/female comparison column, (**E**) whole body row, (**F**) transcript expression table, (**G, H**) file download icons for gene and transcript data, respectively, (**I, J**) interconnected highlighting of gene and transcript data, (**K, L**) in-page help icons for gene and transcript tables, respectively, (**M**) link to UCSC Genome Browser page, and (**N**) icon for pop-up table of links to external sites. These are described in more detail in the text.

The entry form for the ‘Gene’ section is similar to that for FlyAtlas 1 and employs the same custom auto-complete system, which now suppresses the ‘memory’ feature which certain web browsers attempt to apply to the input field. The entry form is repeated on the output page, shown in Figure 1 for a search for *dsx* (A). The default display of total expression of genes is similar to that for FlyAtlas 1, except that there is an additional pair of columns because of the separate analysis of males and females. Figure 1 shows three optional features that may be selected from the ‘tick’ (‘check’) boxes (B): inclusion of standard deviations for the FPKMs (C), ratio of expression in males and females and statistical significance (D), and expression values for whole flies (E).

Because some genes have many different transcripts, it was decided to present the latter in a compact form to aid assimilation of the main features of the results. To this end we have employed a heat-map—rather than numerical—display (F). For users who wish to see the numerical data, this (and the gene data) can be downloaded as tab-separated text files, suitable for importing into spreadsheets such as Microsoft Excel (G, H). To help users locate the transcript expression data corresponding to that for a particular tissue in the Gene table, clicking in the appropriate cell in the latter highlights both sets of cells (I, J). This and the download feature are described in the in-page Help (K, L).

**Figure 2.**
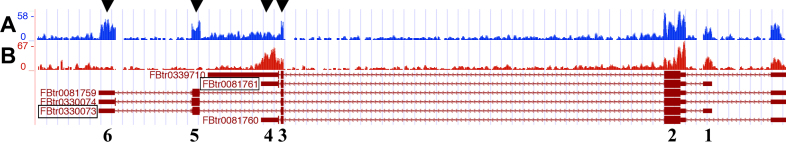
Sequence reads displayed in UCSC Browser. Lanes showing data corresponding to the *dsx* search in Figure 1, but for only two of the tissues: (**A**) male Malpighian tubule, (**B**) female Malpighian tubule. The exons have been numbered, and the names of the two transcripts discussed in the text have been enclosed in boxes for easy identification. Arrow heads indicate the positions in the sequence reads corresponding to exons 3–6. (The gene runs in the 3′ to 5′ direction, as viewed.) The end of a transcript from an adjacent gene has been removed from the map for clarity.

A link out (M) provides a further view of the transcript data in an external window. This view is of a graphic of the raw sequence reads imposed on the physical map of the *Drosophila* genome in the UCSC Browser (12). A detail of this is shown in Figure 2 for the expression of transcripts FBtr0330073 and FBtr0081761 in male and female Malpighian tubules, respectively. It can be seen that the differences between them reflect the celebrated case of the ‘skipping’ of exon 4 of gene *dsx* in males, which plays a key role in sex determination in *Drosophila* (13).

One other feature of Figure 2 should be mentioned: that it contains a transcript (FBtr0081759) absent from the data in FlyAtlas 2, despite its presence in the *Drosophila* Release 6 reference genome. This is an example of one of the ‘missing’ transcripts mentioned above. This transcript (designated dsx-RA on FlyBase maps) is *identical* to transcript FBtr0330074 (dsx-RE). The reason there are two designations for the same transcript here is the demands of nomenclature: inspection of the FlyBase entry for *dsx* (flybase.org/reports/FBgn0000504.html) shows that in addition to the protein, dsx-PA, reflecting the open reading-frame, a second protein, dsx-PE, is translated from this transcript by read-through of the termination codon.

As in FlyAtlas 1, links are provided from the ‘Gene’ table to the listing of the gene in FlyBase (14) and other relevant external sites (N in Figure 1). In addition, one can link to the gene (if present) in FlyAtlas 1, which will continue to be maintained to allow comparison of microarray and RNA-Seq data. Thus, on a sufficiently wide computer display one can view comparable microarray and RNA-Seq data side by side.

The other two types of search in FlyAtlas 2 show only minor changes from FlyAtlas 1. The new ‘Tissue’ search mode is similar to the previous ‘Top’ mode, and allows one to retrieve the genes in a particular tissue showing the greatest absolute or relative expression. It was necessary to provide a separate option for microRNAs as the values for their expression are not comparable quantitatively with those of the genes analysed in the total RNA preparations. The interface for the ‘Category’ search mode is identical to that in FlyAtlas 1(2). In brief, one can search for information on the tissue-specific expression of genes on the basis of either GO ‘name’ (a brief description of the function)—selecting from an autosuggest list, GO identifier, or by freely entered text which will return information for all genes that have been classified with GO ids, the ‘name’ field of which includes the text entered.

The frequency with which the web is accessed using mobile devices has undergone an explosive increase since the release of FlyAtlas 1, and in many areas exceeds access using desktop and laptop computers. Partly because scientific web applications are generally complex, mobile usage is less marked here, and for FlyAtlas 1 was only ∼8% (6.6% mobile, 1.2% tablet) over the last 12 months. Nevertheless we felt that it would be useful to ensure that FlyAtlas 2 was optimized for mobile devices. In addition to implementing ‘responsive’ HTML and CSS (for resizing of content to mobile screen dimensions) we simplified the view on mobiles by changing the text and typeface, suppressing the options to view additional columns and rows in the ‘Gene’ table, and removing the links to external sites. Furthermore, the mobile page was coded to scroll directly to the results table after a search had been run. Together, this allows all or most of a ‘Gene’ results table to appear on a mobile phone in portrait view (Supplementary Figure S3(i)). The Transcript results table was too wide to be accommodated in portrait view but is available in landscape view, and the user is made aware of this (Supplementary Figure S3(ii)).

Occasions may arise in which users of mobile devices would wish to access some of the suppressed functionality available in desktop view. Recent versions of the iPhone Safari and Google Chrome Android web browsers have a function entitled ‘Request Desktop Site’ (or the like), which FlyAtlas 2 has been coded to support (*see* Methods).

## DISCUSSION

Although FlyAtlas 2 is much richer than FlyAtlas 1 in its coverage of specific transcripts and microRNAs in tissues from both male and female flies, it is not without limitations. One is that although microRNAs were specifically included, the methodology did not detect some other smaller RNAs. Furthermore, we are aware of certain genes that give no signal for a particular tissue in FlyAtlas 2, but gave a weak signal in FlyAtlas 1, which was subsequently confirmed by more directed experiments (S. Terhzaz, unpublished). Another limitation is the absence of certain genes and transcripts, although this is generally the result of identical transcripts being given separate designations and reflects the need to complement transcript analysis with proteomic analysis in studying the expression of the corresponding genes.

In order to make our data available to the scientific community as soon as possible FlyAtlas 2 has been released without some of the tissues planned for inclusion, although the labels for these latter were included in the results table (Figure 1) for design purposes. The remaining data should become available in 2018. Work is in progress to integrate FlyAtlas 2 data into FlyBase, adding other tissues as they are completed.

By the criteria of website access and literature citation, FlyAtlas has been an invaluable scientific resource for some years now. We hope that the new features described here will ensure that it continues to be so for many more.

## AVAILABILITY

The FlyAtlas 2 web application is freely accessible to all without registration. A MySQL ‘dump’ of the FlyAtlas 2 database can be downloaded from the ‘Documentation’ page of www.flyatlas2.org. RNA-Seq data have been deposited with European Nucleotide Archive under accession number PRJEB22205 and are now publicly available.

## Supplementary Material

Supplementary Data
