# Supplementary material for: FlyAtlas 2: a new version of the Drosophila melanogaster expression atlas with RNA-Seq, miRNA-Seq and sex-specific data
Source: Nucleic Acids Res. 2017 Oct 24;46(Database issue):D809–15. doi: 10.1093/nar/gkx976 (PMC5753349; doi:10.1093/nar/gkx976)
Supplement: Supplementary Data [file gkx976_supp.pdf]

Supplementary Data

FlyAtlas 2: A new version of the *Drosophila melanogaster* expression atlas with RNA-Seq, miRNA-Seq and sex-specific data

David P. Leader, Sue A. Krause, Aniruddha Pandit, Shireen A. Davies and Julian A. T. Dow

Institute of Molecular Cell and Systems Biology, College of Medical, Veterinary and Life Sciences, University of Glasgow, Glasgow G12 8QQ, UK

Table of Contents

Table S1. Details of *Drosophila* tissues used in FlyAtlas 2
Table S2. Tables in the relational database, FlyAtlas 2 3
Table S3. Validation of FlyAtlas 2 data set by reference to genes with extreme specificity of expression in particular tissues 5
Table S4. Validation of FlyAtlas 2 data set by comparison with results of Fagegaltier *et al*. for most highly enriched microRNAs in testis and ovary 6
Table S5. Comparison of FlyAtlas 2 and FlyAtlas 1 data for 60 most highly expressed genes in the Malpighian tubules 7
Table S6. Genes for which there are no data in FlyAtlas 2 because their transcripts are indistinguishable from those of other genes 11
Table S7. Transcripts for which there are no data in FlyAtlas 2 13
Table S8. RNA genes detected in FlyAtlas 2. 14
Figure S1. Relational database schema for FlyAtlas 2 15
Figure S2. Validation of FlyAtlas 2 data set by reference to genes the transcripts of which show well-characterized sexual dimorphism in soma 16
Figure S3. Responsive adaptation of FlyAtlas 2 search output to mobile devices.....17
References for Supplementary Data 18

Table S1. Number of flies used and yields of total RNA for different tissues

	Adult		Larvae	
**Tissue**	**No. flies**	**RNA (ng/μl)**	**No. flies**	**RNA (ng/μl)**
Whole	10	830	10	1220
Carcass	35	310	15	415
Midgut	80	480	15	310
Hindgut	1100	330	100	220
Anal Pad	1690	202	—	—
Malpighian Tubules	210	430	75	380
Head	10	130*	—	—
Ovary	16	1620	—	—
Testis	60	275	—	—
Accessory Gland	118	310	—	—
Brain	320	210	55	445
Salivary Gland			50	480
Trachea	—	—	230	230
Fat Body	—	—	12	245

*Although this is less than the 200 ng/μl normally required, the RNA passed quality control and yielded satisfactory RNA-Seq data.

Table S2. Tables in the MySQL relational database, FlyAtlas 2

Gene

Field	Type	Null	Key	Default	Extra
FBgn	char(11)	NO	PRI	NULL	
CGNum	varchar(10)	YES		NULL	
Symbol	varchar(40)	YES		NULL	
Name	varchar(100)	YES		NULL	
RomanSymbol	varchar(100)	YES		NULL	
RomanName	varchar(200)	YES		NULL	
Locus	varchar(40)	NO		NULL	
BioType	varchar(25)	NO		NULL	

Ambiguity

Field	Type	Null	Key	Default	Extra
MaskedFBgn	char(11)	NO	PRI	NULL	
MaskingFBgn	char(11)	NO	PRI	NULL	

GeneWithOntol

Field	Type	Null	Key	Default	Extra
FBgn	char(11)	NO	PRI	NULL	
GOid	char(7)	NO	PRI	NULL	

Ontology

Field	Type	Null	Key	Default	Extra
GOid	char(7)	NO	PRI	NULL	
GOname	varchar(250)	NO		NULL	
GOtype	varchar(18)	NO		NULL	

Transcript

Field	Type	Null	Key	Default	Extra
FBgn	char(11)	NO	MUL	NULL	
FBtr	char(11)	NO	PRI	NULL	
TranscriptName	varchar(50)	NO		NULL	
Strand	enum('+','-')	NO		NULL	
ExonCount	int(3)	NO		NULL	
ExonStarts	varchar(1000)	NO		NULL	
ExonEnds	varchar(1000)	NO		NULL	

Tissue

Field	Type	Null	Key	Default	Extra
TissueID	int(3)	NO	PRI	NULL	
Stage	enum('Adult','Larval')	YES		NULL	
Sex	enum('Male','Female','Both')	YES		NULL	
TissueName	varchar(30)	NO		NULL	
Abbreviation	char(2)	NO		NULL	
UniTissueID	int(2)	NO	MUL	NULL	
Reference	enum('Yes','No')	NO		NULL	
Replicates	int(11)	NO		0	

UniTissue

Field	Type	Null	Key	Default	Extra
UniTissueID	int(2)	NO	PRI	NULL	
UniTissueName	varchar(30)	NO		NULL	

GeneFPKM

Field	Type	Null	Key	Default	Extra
FBgn	char(11)	NO	PRI	NULL	
TissueID	int(3)	NO	PRI	NULL	
FPKM	double(8,2)	NO		NULL	
Replicate1	double(8,2)	NO		NULL	
Replicate2	double(8,2)	NO		NULL	
Replicate3	double(8,2)	NO		NULL	
SD	double(8,2)	NO		NULL	

TranscriptFPKM

Field	Type	Null	Key	Default	Extra
FBtr	char(11)	NO	PRI	NULL	
TissueID	int(3)	NO	PRI	NULL	
FPKM	double(8,2)	NO		NULL	
Replicate1	double(8,2)	NO		NULL	
Replicate2	double(8,2)	NO		NULL	
Replicate3	double(8,2)	NO		NULL	
SD	double(8,2)	NO		NULL	

GeneRPM

Field	Type	Null	Key	Default	Extra
FBgn	char(11)	NO	PRI	NULL	
TissueID	int(3)	NO	PRI	NULL	
RPM	int(11)	NO		NULL	
Replicate1	int(11)	NO		NULL	
Replicate2	int(11)	NO		NULL	
Replicate3	int(11)	NO		NULL	
SD	int(11)	NO		NULL	

TranscriptRPM

Field	Type	Null	Key	Default	Extra
FBtr	char(11)	NO	PRI	NULL	
TissueID	int(3)	NO	PRI	NULL	
RPM	int(11)	NO		NULL	
Replicate1	int(11)	NO		NULL	
Replicate2	int(11)	NO		NULL	
Replicate3	int(11)	NO		NULL	
SD	int(11)	NO		NULL	

Table S3. Validation of FlyAtlas 2 data set by reference to genes with extreme specificity of expression in particular tissues

Gene	Head	Midgut	Malpighian Tubules	Hindgut	Ovary	Testis
**trp**	**134**<br>**2650**	0.1<br>0	0<br>0	0<br>0	0<br>0	0<br>0
**Jon99Ci**	0<br>12	**1998**<br>**6335**	11.4<br>0	7.6<br>0	0<br>0	0.4<br>11
**CG15408**	0<br>0	0.6<br>0	**597**<br>**4338**	0.1<br>0	0<br>0	2.2<br>7
**CG9993**	0<br>0	0.2<br>0.1	2.4<br>1	**595**<br>**2648**	0.1<br>0	0.2<br>0
**otu**	0<br>0	0.2<br>8	0.8<br>0	0.5<br>0	**59**<br>**1353**	1.4<br>6
**Mst84Dd**	1.1<br>0	0.1<br>0	0.3<br>0	13<br>0	1<br>0	**4997**<br>**2526**

Red: FlyAtlas 2; **Blue**: FlyAtlas 1 (1)

Units are arbitrary signal units in the case of FlyAtlas 1, and FPKM in the case of FlyAtlas 2.

Table S4. Validation of FlyAtlas 2 data set by comparison with results of Fagegaltier *et al.*(2) for the most highly enriched microRNAs in testis and ovary

	**Fagegaltier *et al.***			**This study**		
**mir**	**Testis**	**Testis/Total**	**Rank**	**Testis**	**Testis/Total**	**Rank**
mir-975	7469	1.00	1	2218	0.98	na
mir-310	11746	1.00	2	7931	0.97	8
mir-311	12537	0.99	3	56554	0.98	5
mir-959	60382	0.99	4	7035	0.98	7
mir-978	29600	0.99	5	495	0.99	na
mir-964	35403	0.99	6	5035	0.98	na
mir-976	26338	0.99	7	13366	0.98	3
mir-312	5964	0.98	8	46104	0.98	4
mir-977	28350	0.98	9	294	0.98	na
mir-983	23562	0.97	10	1367	0.97	na
mir-988	23055	0.96	11	546	0.84	na
mir-984	7704	0.93	12	1643	0.98	na
mir-960	19026	0.92	13	20604	0.99	2
mir-12	217798	0.91	14	27109	0.83	11
mir-274	27748	0.89	15	4689	0.63	na
mir-317	15712	0.87	16	74325	0.57	22
mir-9b	23545	0.85	17	3956	0.89	na
mir-305	65041	0.82	18	11126	0.65	na
mir-34	40124	0.81	19	13599	0.46	24
mir-316	15460	0.74	20	4022	0.79	na
mir-11	32372	0.69	21	20971	0.61	21
mir-125	11659	0.69	22	2108	0.62	na

	**Fagegaltier *et al.***			**This study**		
**mir**	**Ovary**	**Ovary/Total**	**Rank**	**Ovary**	**Ovary/Total**	**Rank**
mir-318	34886	1.00	1	122908	0.76	13
mir-994	14463	0.99	2	18545	0.82	12
mir-275	12586	0.94	3	10347	0.83	10
mir-989	88414	0.87	4	20745	0.88	1
mir-9b	10570	0.87	5	9038	0.85	4
mir-184	30821	0.80	6	152506	0.65	18
mir-996	28482	0.78	7	3193	0.73	na
mir-79	6723	0.75	8	90	0.79	na
mir-33	4025	0.67	9	12173	0.59	22
mir-34	6344	0.66	10	1744	0.16	na

na = not applicable as signal < 5% max

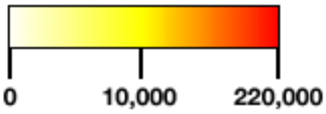

The absolute values for expression of transcripts are not comparable between the two studies although each employs the three-colour scale, shown.

Table S5. Comparison of FlyAtlas 2 and FlyAtlas 1 data for 60 most highly expressed genes in the Malpighian tubules
(i) Ordered by most enriched genes in FlyAtlas 1

	FlyAtlas 1			FlyAtlas 2		
Gene	Abundance	Whole	Enrich.	FPKM	Whole	Enrich.
CG4314	4177	45	93	190	4.7	40
CG9270	1106	13	81	54	1.3	42
CG33282	798	10	73	103	1.3	79
CG15088	918	13	68	95	5.1	19
CG18095	980	14	68	271	4.6	59
CG13905	1534	22	67	289	3.7	78
CG3212	281	4	64	40	0.7	57
CG15408	4337	67	64	597	13	46
CG13311	1876	29	63	428	9.3	46
CG8837	1088	17	61	101	3.7	27
CG10207	2613	43	60	946	17.9	53
CG8028	2008	34	59	212	5	42
CG8620	979	16	59	156	2.4	65
CG17751	1859	31	58	196	3.4	58
CG31792	218	3	58	13	0.3	43
CG1139	1727	30	58	116	7.8	15
CG18473	4250	75	56	297	7.1	42
CG16727	2501	44	56	283	4.6	62
CG9717	1989	37	53	113	5.4	21
CG3285	3501	67	52	364	8.9	41
CG33281	1936	39	49	131	6	22
CG42235	1425	29	49	1542	29.5	52
CG13977	2635	54	49	437	10.2	43
CG6465	3646	78	46	226	5.8	39
CG15221	594	13	45	62	1.7	36
CG34043	4207	94	44	825	11.1	74
CG15155	5291	120	44	1695	33	51
CG10369	4932	115	43	418	10	42
CG3270	6215	147	42	903	22.5	40
CG14606	1468	34	42	276	5.6	49
CG14575	512	12	42	49	1.1	45
CG6602	3426	81	42	805	15	54
CG17752	6340	156	40	1131	18	63
CG17110	1174	29	40	91	1.6	57
CG2759	1571	39	40	127	4.3	30
CG31272	6019	151	40	533	8.4	63
CG30277	1637	42	39	212	4.3	49
CG31106	5145	133	38	710	12.2	58
CG31663	1365	36	38	210	5.8	36
CG34456	303	8	38	67	2	34
CG32024	2491	67	37	593	10.3	58
CG6125	4530	122	37	319	8.3	38
CG17027	1526	41	37	210	6.8	31
CG5697	3029	82	37	302	8.3	36
CG32024	2492	67	37	593	10.3	58
CG10626	348	9	36	22	0.8	28

CG32023	4818	133	36	1000	21	48
CG15556	1187	33	36	69	4.6	15
CG7171	6590	183	36	940	9.4	100
CG10006	902	25	36	365	7.3	50
CG32843	992	28	35	45	1.8	25
CG9702	2377	67	35	223	8	28
CG30016	3945	112	35	1503	45	33
CG31380	948	27	34	112	4	28
CG3690	566	16	34	356	6.7	53
CG14957	1989	60	33	312	5.2	60
CG2187	2332	72	32	20	0.7	29
CG9444	398	12	32	31	0.7	44
CG5361	535	16	32	49	0.8	61
CG4484	1386	44	31	133	6.4	21
CG31097	374	19	20	101	1.5	67
CG33258	6982	383	18	1051	15.7	67
CG9259	7558	370	19	2756	49	56
CG2196	7242	275	26	1854	33	56
CG11892	7339	506	14	2885	52	55
CG10514	6203	438	14	1690	31	55
CG15406	4941	165	30	1106	20.5	54
CG3290	5919	314	19	856	16	54
CG6726	8244	585	14	1912	36	53
CG17636	811	26	31	119	2.4	50
CG7882	3485	129	27	1010	21	48
CG42807	7441	1112	7	1680	35	48
CG2680	3243	110	30	747	15.8	47
CG17522	3138	114	28	434	9.2	47
CG8791	2754	98	28	178	3.8	47
CG3168	7340	734	10	3617	82	44
CG7874	6383	1213	5	5921	136	44
CG10513	6469	309	21	1172	28	42
CG3380	6251	227	27	582	14	42
CG14857	182	8	21	90	2.2	41
CG13309	5652	214	26	3051	75	41

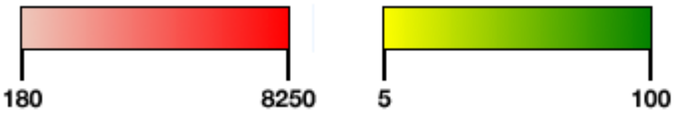

The abundance values for FlyAtlas 1 are in arbitrary units. The 'Enrich.' column is the enrichment calculated by dividing the absolute value of expression in Malpighian tubules by that in whole flies.

(ii) Ordered by most enriched genes in FlyAtlas 2

	FlyAtlas 1			FlyAtlas 2		
Gene	Abundance	Whole	Enrich.	FPKM	Whole	Enrich.
CG7171	6590	183	36	940	9.4	100
CG33282	798	10	73	103	1.3	79
CG13905	1534	22	67	289	3.7	78
CG34043	4207	94	44	825	11.1	74
CG8620	979	16	59	156	2.4	65
CG31272	6019	151	40	533	8.4	63
CG17752	6340	156	40	1131	18	63
CG16727	2501	44	56	283	4.6	62
CG5361	535	16	32	49	0.8	61
CG14957	1989	60	33	312	5.2	60
CG18095	980	14	68	271	4.6	59
CG31106	5145	133	38	710	12.2	58
CG17751	1859	31	58	196	3.4	58
CG32024	2491	67	37	593	10.3	58
CG32024	2492	67	37	593	10.3	58
CG3212	281	4	64	40	0.7	57
CG17110	1174	29	40	91	1.6	57
CG6602	3426	81	42	805	15	54
CG3690	566	16	34	356	6.7	53
CG10207	2613	43	60	946	17.9	53
CG42235	1425	29	49	1542	29.5	52
CG15155	5291	120	44	1695	33	51
CG10006	902	25	36	365	7.3	50
CG30277	1637	42	39	212	4.3	49
CG14606	1468	34	42	276	5.6	49
CG32023	4818	133	36	1000	21	48
CG13311	1876	29	63	428	9.3	46
CG15408	4337	67	64	597	13	46
CG14575	512	12	42	49	1.1	45
CG9444	398	12	32	31	0.7	44
CG31792	218	3	58	13	0.3	43
CG13977	2635	54	49	437	10.2	43
CG8028	2008	34	59	212	5	42
CG18473	4250	75	56	297	7.1	42
CG10369	4932	115	43	418	10	42
CG9270	1106	13	81	54	1.3	42
CG3285	3501	67	52	364	8.9	41
CG4314	4177	45	93	190	4.7	40
CG3270	6215	147	42	903	22.5	40
CG6465	3646	78	46	226	5.8	39
CG6125	4530	122	37	319	8.3	38
CG15221	594	13	45	62	1.7	36
CG5697	3029	82	37	302	8.3	36
CG31663	1365	36	38	210	5.8	36
CG34456	303	8	38	67	2	34
CG30016	3945	112	35	1503	45	33
CG17027	1526	41	37	210	6.8	31
CG2759	1571	39	40	127	4.3	30

CG2187	2332	72	32	20	0.7	29
CG31380	948	27	34	112	4	28
CG9702	2377	67	35	223	8	28
CG10626	348	9	36	22	0.8	28
CG8837	1088	17	61	101	3.7	27
CG32843	992	28	35	45	1.8	25
CG33281	1936	39	49	131	6	22
CG9717	1989	37	53	113	5.4	21
CG4484	1386	44	31	133	6.4	21
CG15088	918	13	68	95	5.1	19
CG15556	1187	33	36	69	4.6	15
CG1139	1727	30	58	116	7.8	15
CG17636	811	26	31	119	2.4	50
CG15406	4941	165	30	1106	20.5	54
CG2680	3243	110	30	747	15.8	47
CG17522	3138	114	28	434	9.2	47
CG8791	2754	98	28	178	3.8	47
CG7882	3485	129	27	1010	21	48
CG3380	6251	227	27	582	14	42
CG2196	7242	275	26	1854	33	56
CG13309	5652	214	26	3051	75	41
CG10513	6469	309	21	1172	28	42
CG14857	182	8	21	90	2.2	41
CG31097	374	19	20	101	1.5	67
CG9259	7558	370	19	2756	49	56
CG3290	5919	314	19	856	16	54
CG33258	6982	383	18	1051	15.7	67
CG11892	7339	506	14	2885	52	55
CG10514	6203	438	14	1690	31	55
CG6726	8244	585	14	1912	36	53
CG3168	7340	734	10	3617	82	44
CG42807	7441	1112	7	1680	35	48
CG7874	6383	1213	5	5921	136	44

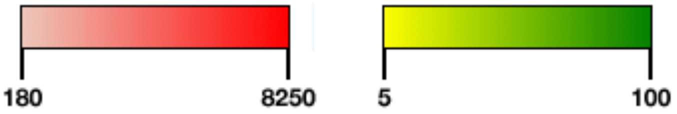

The abundance values for FlyAtlas 1 are in arbitrary units. The ‘Enrich.’ column is the enrichment calculated by dividing the absolute value of expression in Malpighian tubules by that in whole flies.

Table S6. Genes for which there are no data in FlyAtlas 2 because their transcripts are indistinguishable from those of other genes

Masked Gene	Symbol	Masking Gene	Symbol
FBgn0003067	Pepck	FBgn0266488	CG45087
FBgn0004407	DNApol-γ35	FBgn0064115	GatC
FBgn0024182	waw	FBgn0024251	bbx
FBgn0025355	SuUR	FBgn0266540	CG45101
FBgn0025825	HDAC3	FBgn0266539	CG45100
FBgn0026778	Rad1	FBgn0085343	Sws1
FBgn0027358	Tim9b	FBgn0031041	CG12788
FBgn0028581	mei-217	FBgn0002709	mei-218
FBgn0029154	Menl-1	FBgn0029153	Menl-2
FBgn0030298	Or10a	FBgn0045502	Gr10a
FBgn0031159	CG11566	FBgn0064123	stg1
FBgn0031454	CG9960	FBgn0031455	Snapin
FBgn0032755	CG17344	FBgn0263982	CG43731
FBgn0033693	CG13175	FBgn0053964	CG33964
FBgn0034649	PIG-M	FBgn0259726	CG42380
FBgn0039367	CG17197	FBgn0039366	CG17198
FBgn0040715	CG15386	FBgn0259717	CG42371
FBgn0042182	CG18749	FBgn0064126	CG33722
FBgn0050072	Obp50c	FBgn0050073	Obp50b
FBgn0050080	CG30080	FBgn0261538	CG42662
FBgn0050100	CG30100	FBgn0259718	CG42372
FBgn0050481	mRpL53	FBgn0053155	CG33155
FBgn0052298	CG32298	FBgn0052299	CG32299
FBgn0061361	CG33786	FBgn0061362	CG33785
FBgn0085206	CG34177	FBgn0085207	CG34178
FBgn0085344	CG34315	FBgn0005654	lat
FBgn0085346	CG34317	FBgn0260468	CG7950
FBgn0250911	CG42245	FBgn0035357	MEP-1
FBgn0259199	Snup	FBgn0259200	CG42304
FBgn0259202	CG42306	FBgn0034372	Gint3
FBgn0259709	CG42363	FBgn0259708	CG42362
FBgn0259721	CG42375	FBgn0260464	CG9288
FBgn0259725	CG42379	FBgn0259726	CG42380
FBgn0259727	CG42381	FBgn0259726	CG42380
FBgn0259730	tal-1A	FBgn0259733	tal-AA
FBgn0259731	tal-2A	FBgn0259733	tal-AA
FBgn0259732	tal-3A	FBgn0259733	tal-AA
FBgn0259933	CG42456	FBgn0035436	CG12016
FBgn0259990	CG42487	FBgn0039565	CG4884
FBgn0259991	CG42488	FBgn0039339	CG5116
FBgn0260222	CG42496	FBgn0050290	Ppcdc
FBgn0260223	CG42497	FBgn0027360	Tim10

FBgn0260224	CG42498	FBgn0039405	PIG-P
FBgn0260228	CG42502	FBgn0040992	CG10570
FBgn0260229	CG42503	FBgn0039280	Mocs2
FBgn0260231	CG42505	FBgn0260230	CG42504
FBgn0260237	CG42511	FBgn0037689	CG8135
FBgn0260401	MED9	FBgn0260392	CG42518
FBgn0260454	CG17139	FBgn0260453	CG17140
FBgn0260759	CG42557	FBgn0260760	CG42558
FBgn0261353	Ccdc56	FBgn0261381	mtTFB1
FBgn0261357	CG42634	FBgn0261358	CG42635
FBgn0261536	CG42660	FBgn0261537	CG42661
FBgn0261679	CG42726	FBgn0261680	CG42727
FBgn0261683	B9d2	FBgn0261697	tectonic
FBgn0261704	CG42740	FBgn0027544	CG2217
FBgn0261837	pre-mod(mdg4)-T	FBgn0261838	pre-mod(mdg4)-Z
FBgn0262837	CG43201	FBgn0262807	CG43178
FBgn0263387	CG43439	FBgn0250849	CG32388
FBgn0264298	CG43776	FBgn0264297	CG43775
FBgn0264364	CG43816	FBgn0045827	CG13245
FBgn0265083	CG44194	FBgn0038107	CG17327
FBgn0265968	Tfb5	FBgn0031668	CG31917
FBgn0266195	Tgs1	FBgn0261019	moi
FBgn0266435	CG45065	FBgn0266434	CG45064
FBgn0266438	PIG-Z	FBgn0266439	CG45069
FBgn0266445	CG45075	FBgn0266450	Kr-h1
FBgn0266489	CG45088	FBgn0027581	CG6191
FBgn0266525	CG45092	FBgn0033095	CG3409
FBgn0266526	CG45093	FBgn0050497	CG30497
FBgn0266538	CG45099	FBgn0038927	CG6015

‘Masked’ Genes are those that were absent from the FPKM or RPM output of the computational analysis of the sequence reads. In each case an overlapping gene was identified with an identical transcript and this is referred to as a ‘masking’ gene and is listed in the second half of the table.

Table S7. Transcripts for which there are no data in FlyAtlas 2

FBtr0070244	FBtr0083933	FBtr0100678	FBtr0301963	FBtr0330088	FBtr0330620	FBtr0344301
FBtr0070691	FBtr0084032	FBtr0100836	FBtr0302020	FBtr0330091	FBtr0330627	FBtr0344322
FBtr0070742	FBtr0084123	FBtr0110903	FBtr0302021	FBtr0330094	FBtr0330629	FBtr0344372
FBtr0071008	FBtr0084161	FBtr0110936	FBtr0302194	FBtr0330095	FBtr0330631	FBtr0344424
FBtr0071052	FBtr0084197	FBtr0110984	FBtr0302271	FBtr0330096	FBtr0330632	FBtr0344430
FBtr0071140	FBtr0084211	FBtr0112368	FBtr0302275	FBtr0330097	FBtr0330709	FBtr0344439
FBtr0071141	FBtr0084509	FBtr0112510	FBtr0302512	FBtr0330102	FBtr0330713	FBtr0344448
FBtr0071361	FBtr0084522	FBtr0112516	FBtr0302553	FBtr0330108	FBtr0330734	FBtr0344452
FBtr0071660	FBtr0084668	FBtr0112561	FBtr0302557	FBtr0330114	FBtr0331155	FBtr0344455
FBtr0071680	FBtr0084837	FBtr0112576	FBtr0302558	FBtr0330115	FBtr0331156	FBtr0344459
FBtr0071681	FBtr0084838	FBtr0112596	FBtr0303038	FBtr0330120	FBtr0331158	FBtr0344460
FBtr0072267	FBtr0084897	FBtr0112744	FBtr0303074	FBtr0330121	FBtr0331160	FBtr0344466
FBtr0072290	FBtr0085088	FBtr0112745	FBtr0303078	FBtr0330123	FBtr0331161	FBtr0344468
FBtr0072657	FBtr0085385	FBtr0112945	FBtr0303083	FBtr0330137	FBtr0331162	FBtr0344489
FBtr0072758	FBtr0085474	FBtr0113037	FBtr0303201	FBtr0330140	FBtr0331167	FBtr0344518
FBtr0072887	FBtr0085507	FBtr0113157	FBtr0303202	FBtr0330146	FBtr0331171	FBtr0344522
FBtr0073199	FBtr0085621	FBtr0113373	FBtr0303223	FBtr0330148	FBtr0331181	FBtr0344526
FBtr0073279	FBtr0085694	FBtr0113717	FBtr0303433	FBtr0330150	FBtr0331182	FBtr0344528
FBtr0073485	FBtr0085701	FBtr0114466	FBtr0303603	FBtr0330152	FBtr0331183	FBtr0344529
FBtr0073731	FBtr0085760	FBtr0273378	FBtr0303848	FBtr0330154	FBtr0331251	FBtr0344540
FBtr0073803	FBtr0085821	FBtr0273438	FBtr0304686	FBtr0330157	FBtr0331271	FBtr0344544
FBtr0074008	FBtr0086450	FBtr0273450	FBtr0305003	FBtr0330163	FBtr0331298	FBtr0344557
FBtr0074034	FBtr0086569	FBtr0290204	FBtr0305480	FBtr0330165	FBtr0331300	FBtr0344559
FBtr0074298	FBtr0086701	FBtr0290283	FBtr0305666	FBtr0330168	FBtr0331335	FBtr0344560
FBtr0074412	FBtr0086780	FBtr0290322	FBtr0305796	FBtr0330170	FBtr0331338	FBtr0344561
FBtr0074736	FBtr0087125	FBtr0290341	FBtr0305907	FBtr0330171	FBtr0331339	FBtr0344565
FBtr0074738	FBtr0087140	FBtr0299683	FBtr0305948	FBtr0330177	FBtr0331501	FBtr0344566
FBtr0074767	FBtr0087141	FBtr0299686	FBtr0306076	FBtr0330178	FBtr0331502	FBtr0344567
FBtr0074991	FBtr0087319	FBtr0299688	FBtr0306107	FBtr0330179	FBtr0331503	FBtr0344568
FBtr0075203	FBtr0087383	FBtr0299689	FBtr0306142	FBtr0330182	FBtr0331507	FBtr0344621
FBtr0075518	FBtr0087462	FBtr0299691	FBtr0306748	FBtr0330186	FBtr0331510	FBtr0344622
FBtr0075525	FBtr0087678	FBtr0299692	FBtr0306911	FBtr0330188	FBtr0331603	FBtr0344632
FBtr0075707	FBtr0087755	FBtr0299695	FBtr0306913	FBtr0330190	FBtr0332159	FBtr0344651
FBtr0075832	FBtr0087767	FBtr0299802	FBtr0306975	FBtr0330192	FBtr0332229	FBtr0344715
FBtr0075890	FBtr0087786	FBtr0299803	FBtr0307877	FBtr0330201	FBtr0332230	FBtr0345028
FBtr0075911	FBtr0087829	FBtr0299805	FBtr0308123	FBtr0330202	FBtr0332466	FBtr0345323
FBtr0075989	FBtr0087830	FBtr0299807	FBtr0308135	FBtr0330203	FBtr0332566	FBtr0345349
FBtr0076203	FBtr0088263	FBtr0299809	FBtr0308494	FBtr0330206	FBtr0332867	FBtr0345384
FBtr0076348	FBtr0088356	FBtr0299817	FBtr0308891	FBtr0330208	FBtr0333420	FBtr0345441
FBtr0076352	FBtr0088368	FBtr0299820	FBtr0309074	FBtr0330209	FBtr0333502	FBtr0345444
FBtr0076944	FBtr0088411	FBtr0299951	FBtr0309271	FBtr0330217	FBtr0334121	FBtr0345460
FBtr0077027	FBtr0088435	FBtr0299952	FBtr0309790	FBtr0330230	FBtr0334122	FBtr0345499
FBtr0077054	FBtr0088499	FBtr0299962	FBtr0309800	FBtr0330236	FBtr0334123	FBtr0345703
FBtr0077164	FBtr0088566	FBtr0299976	FBtr0309833	FBtr0330238	FBtr0334124	FBtr0345940
FBtr0077747	FBtr0088581	FBtr0299989	FBtr0310337	FBtr0330240	FBtr0334125	FBtr0346040
FBtr0077780	FBtr0088686	FBtr0299990	FBtr0310498	FBtr0330249	FBtr0334126	FBtr0346392
FBtr0077810	FBtr0089477	FBtr0299991	FBtr0329859	FBtr0330252	FBtr0334127	FBtr0346393
FBtr0077842	FBtr0089481	FBtr0299997	FBtr0329869	FBtr0330254	FBtr0335048	FBtr0346453
FBtr0077941	FBtr0089555	FBtr0299998	FBtr0329893	FBtr0330283	FBtr0335411	FBtr0346505
FBtr0078426	FBtr0091475	FBtr0299999	FBtr0329924	FBtr0330287	FBtr0336814	FBtr0346507
FBtr0078767	FBtr0091627	FBtr0300239	FBtr0329935	FBtr0330288	FBtr0337006	FBtr0346513
FBtr0079004	FBtr0091650	FBtr0300240	FBtr0329939	FBtr0330292	FBtr0339086	FBtr0346514
FBtr0079300	FBtr0091694	FBtr0300287	FBtr0329945	FBtr0330293	FBtr0339299	FBtr0346522
FBtr0079733	FBtr0091702	FBtr0300437	FBtr0329946	FBtr0330310	FBtr0339351	FBtr0346525
FBtr0079890	FBtr0091704	FBtr0300439	FBtr0329947	FBtr0330315	FBtr0339429	FBtr0346588
FBtr0080133	FBtr0091710	FBtr0300440	FBtr0329951	FBtr0330322	FBtr0339457	FBtr0346628
FBtr0080134	FBtr0091716	FBtr0300467	FBtr0329953	FBtr0330327	FBtr0339458	FBtr0346704
FBtr0080542	FBtr0091747	FBtr0300494	FBtr0329957	FBtr0330333	FBtr0339531	FBtr0346728
FBtr0080947	FBtr0091757	FBtr0300624	FBtr0329966	FBtr0330335	FBtr0339540	FBtr0346757
FBtr0081002	FBtr0091787	FBtr0300625	FBtr0329971	FBtr0330337	FBtr0339551	FBtr0346762
FBtr0081040	FBtr0091794	FBtr0300626	FBtr0329980	FBtr0330341	FBtr0339667	FBtr0346763
FBtr0081043	FBtr0091936	FBtr0300635	FBtr0329984	FBtr0330360	FBtr0339676	FBtr0346765
FBtr0081102	FBtr0100026	FBtr0300637	FBtr0329988	FBtr0330371	FBtr0340022	FBtr0346792
FBtr0081152	FBtr0100050	FBtr0300638	FBtr0329992	FBtr0330373	FBtr0340303	FBtr0346798
FBtr0081440	FBtr0100143	FBtr0300643	FBtr0329993	FBtr0330376	FBtr0340367	FBtr0346863
FBtr0081759	FBtr0100187	FBtr0300646	FBtr0329994	FBtr0330378	FBtr0340393	FBtr0346865
FBtr0081956	FBtr0100190	FBtr0300651	FBtr0330012	FBtr0330380	FBtr0340487	FBtr0346866
FBtr0082151	FBtr0100253	FBtr0300661	FBtr0330028	FBtr0330382	FBtr0342717	FBtr0346907
FBtr0082177	FBtr0100285	FBtr0300664	FBtr0330032	FBtr0330383	FBtr0342958	FBtr0346949
FBtr0082554	FBtr0100323	FBtr0300900	FBtr0330033	FBtr0330384	FBtr0343134	FBtr0346951
FBtr0082603	FBtr0100383	FBtr0301017	FBtr0330036	FBtr0330386	FBtr0343221	FBtr0346955
FBtr0082914	FBtr0100445	FBtr0301130	FBtr0330039	FBtr0330387	FBtr0343439	FBtr0346958
FBtr0082977	FBtr0100471	FBtr0301256	FBtr0330040	FBtr0330388	FBtr0343554	FBtr0346959
FBtr0083228	FBtr0100472	FBtr0301258	FBtr0330046	FBtr0330395	FBtr0343791	FBtr0346998
FBtr0083261	FBtr0100495	FBtr0301274	FBtr0330050	FBtr0330402	FBtr0343835	FBtr0347000
FBtr0083435	FBtr0100536	FBtr0301427	FBtr0330058	FBtr0330408	FBtr0344069	FBtr0347001
FBtr0083584	FBtr0100545	FBtr0301436	FBtr0330059	FBtr0330413	FBtr0344072	FBtr0347004
FBtr0083590	FBtr0100591	FBtr0301669	FBtr0330068	FBtr0330414	FBtr0344098	FBtr0347006
FBtr0083677	FBtr0100609	FBtr0301700	FBtr0330070	FBtr0330597	FBtr0344100	FBtr0347011
FBtr0083752	FBtr0100619	FBtr0301701	FBtr0330078	FBtr0330604	FBtr0344112	FBtr0347067
FBtr0083762	FBtr0100647	FBtr0301869	FBtr0330080	FBtr0330605	FBtr0344180	FBtr0347349
FBtr0083912	FBtr0100655	FBtr0301951	FBtr0330081	FBtr0330608	FBtr0344198	

Table S8. RNA genes detected in FlyAtlas 2

Type	Total	Signal
All RNA genes	3603	1828
pseudogene	232	74
ncRNA	549	370
snRNA	31	24
tRNA	314	8
rRNA	151	6
snoRNA	288	214
lincRNA	1800	1003
pre miRNA	238	129

A signal was regarded as an FPKM greater than 2 or an RPM (microRNAs) greater than 100 in at least one tissue.

Figure S1. Relational database schema for FlyAtlas 2

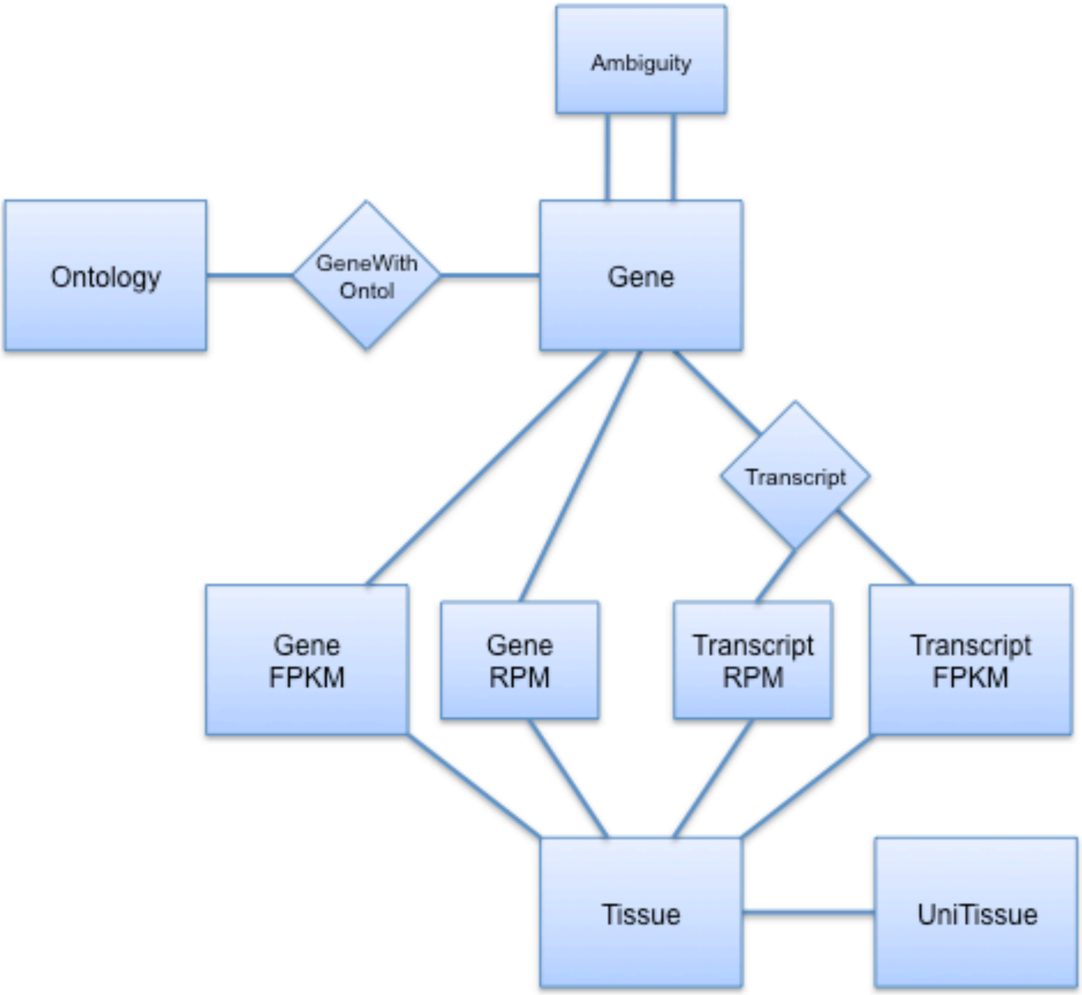

Figure S2. Validation of FlyAtlas 2 data set by reference to genes the transcripts of which show well-characterized sexual dimorphism in soma

tra

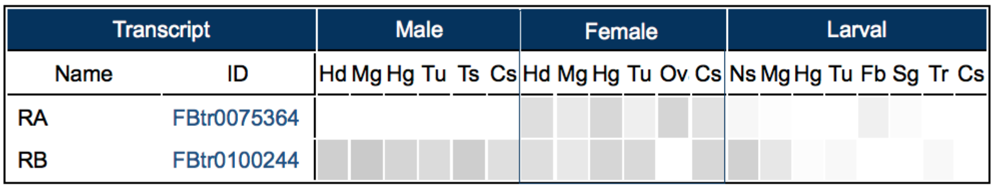

The isoform FBtr0075364, expressed only in females, is the key factor allowing splicing of exon 4 of *dsx* in females to produce *dsx* transcript FBtr008176 (3).

dsx

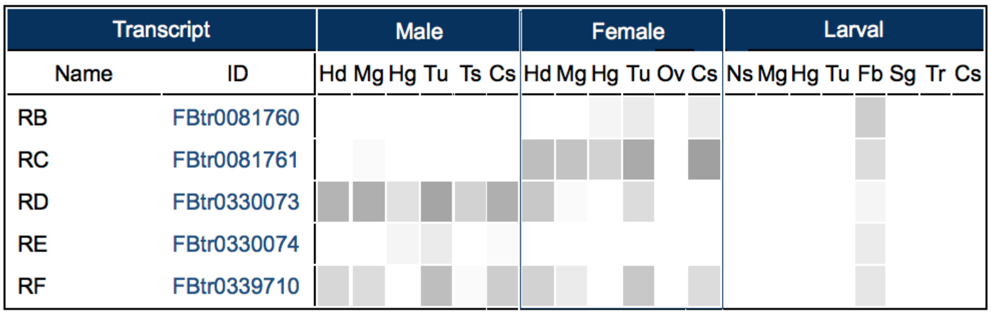

The differential splicing of exon 4 causes FBtr0081761 to be expressed only in females, and FBtr0330073 in males, as explained in the main text.

fru

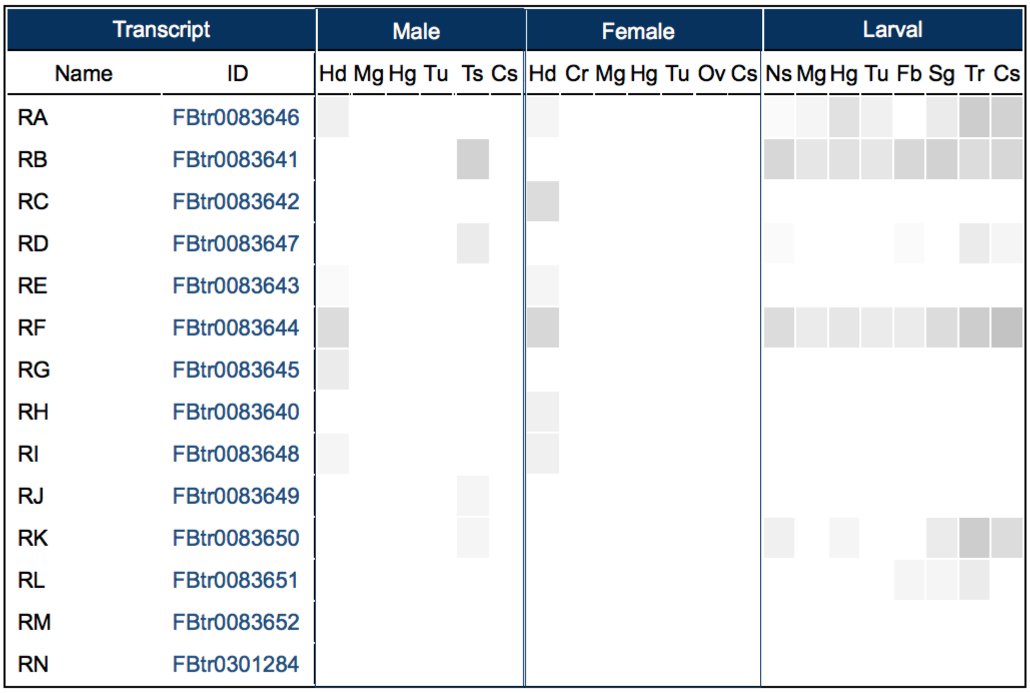

Differences in exons 2 and 3 resulting from alternative splicing results in male-specific and female-specific forms of *fru*, the male-specific ones being implicated in control of courtship behaviour (4). The isoform here found only in the heads of males, FBtr0083645, shows the male-specific splice, whereas FBtr0083642, here found only in females, shows the female-specific splice.

Figure S3. Responsive adaptation of FlyAtlas 2 search output to mobile devices

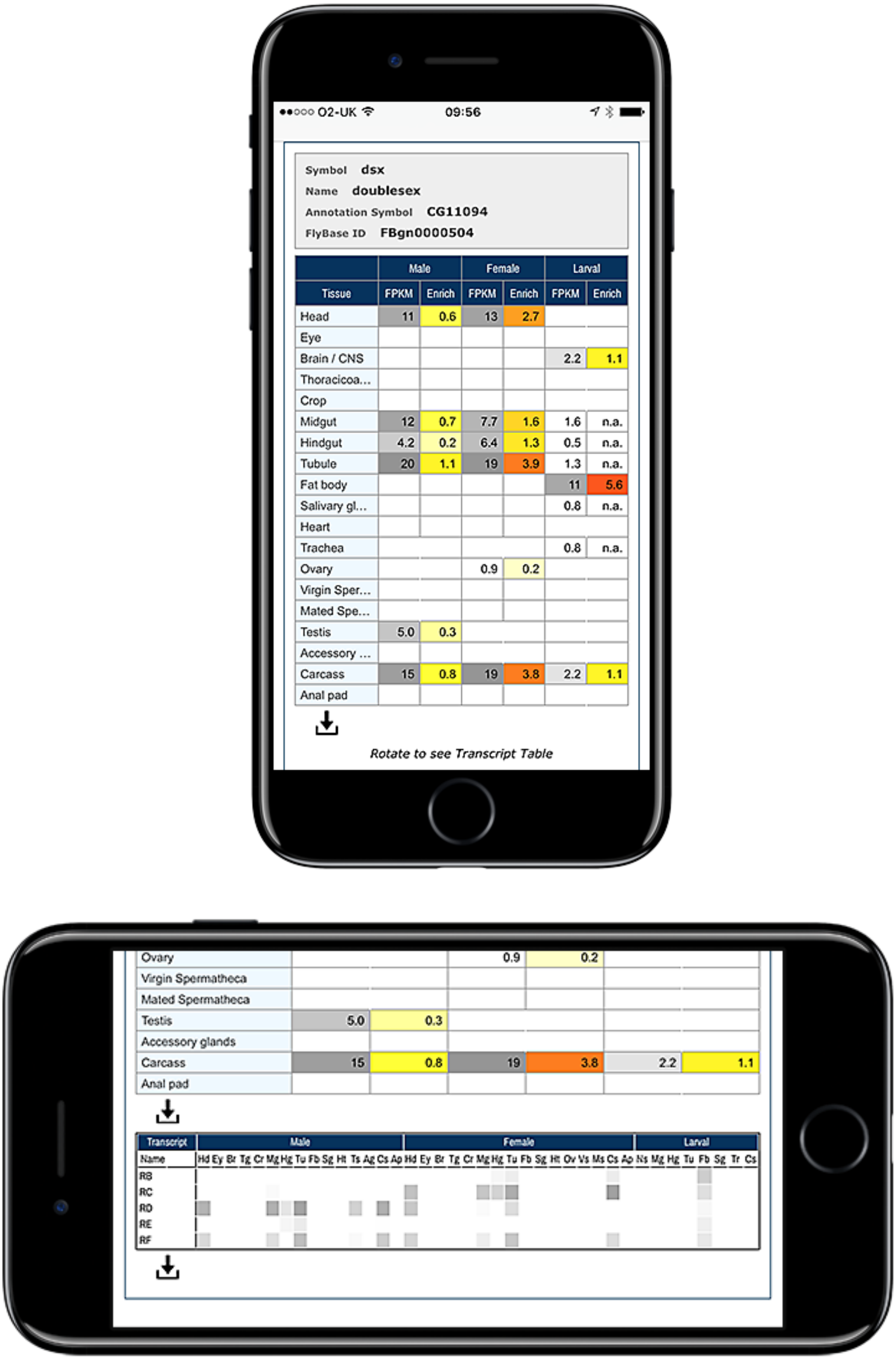

An iPhone 7 (375×667 px) is illustrated. On an iPhone 5 (320×568 px) in portrait view the table is visible, but not the area below it.

References for Supplementary Data

1. Chintapalli, V.R., Wang, J. and Dow, J.A.T. (2007) Using FlyAtlas to identify better Drosophila melanogaster models of human disease. *Nat Genet*, **39**, 715-720.
2. Fagegaltier, D., König, A., Gordon, A., Lai, E.C., Gingeras, T.R., Hannon, G.J. and Shcherbata, H.R. (2014) A genome-wide survey of sexually dimorphic expression of Drosophila miRNAs identifies the steroid hormone-induced miRNA let-7 as a regulator of sexual identity. *Genetics*, **198**, 647-668.
3. Lynch, K.W. and Maniatis, T. (1996) Assembly of specific SR protein complexes on distinct regulatory elements of the Drosophila doublesex splicing enhancer. *Genes & Development*, **10**, 2089-2101.
4. Ryner, L.C., Goodwin, S.F., Castrillon, D.H., Anand, A., Villella, A., Baker, B.S., Hall, J.C., Taylor, B.J. and Wasserman, S.A. (1996) Control of male sexual behavior and sexual orientation in Drosophila by the fruitless gene. *Cell*, **87**, 1079-1089.
